# A fluorogenic micrococcal nuclease-based probe for fast detection and optical imaging of *Staphylococcus aureus* in prosthetic joint and fracture-related infections

**DOI:** 10.1007/s00259-023-06499-4

**Published:** 2023-11-14

**Authors:** Jorrit W.A. Schoenmakers, Marina López‑Álvarez, Frank F.A. IJpma, Marjan Wouthuyzen-Bakker, James O. McNamara, Marleen van Oosten, Paul C. Jutte, Jan Maarten van Dijl

**Affiliations:** 1 Department of Medical Microbiology and Infection Prevention, University of Groningen, University Medical Center Groningen (UMCG), Hanzeplein 1, 9700RB Groningen, The Netherlands; 2 Department of Orthopaedics, University of Groningen, University Medical Center Groningen (UMCG), Groningen, The Netherlands; 3 Department of Surgery, Division of Trauma Surgery, University of Groningen (UMCG), Groningen, The Netherlands; 4 Nuclease Probe Technologies, Inc, Lowell, MA USA

**Keywords:** Infection imaging, Optical probe, Orthopedic infection, Periprosthetic joint infection, Fracture-related infection, *Staphylococcus aureus*, Micrococcal nuclease

## Abstract

**Purpose:**

*Staphylococcus aureus* is the most common and impactful multi-drug resistant pathogen implicated in (periprosthetic) joint infections (PJI) and fracture-related infections (FRI). Therefore, the present proof-of-principle study was aimed at the rapid detection of *S. aureus* in synovial fluids and biofilms on extracted osteosynthesis materials through bacteria-targeted fluorescence imaging with the ‘smart-activatable’ DNA-based AttoPolyT probe. This fluorogenic oligonucleotide probe yields large fluorescence increases upon cleavage by micrococcal nuclease, an enzyme secreted by *S. aureus*.

**Methods:**

Synovial fluids from patients with suspected PJI and extracted osteosynthesis materials from trauma patients with suspected FRI were inspected for *S. aureus* nuclease activity with the AttoPolyT probe. Biofilms on osteosynthesis materials were imaged with the AttoPolyT probe and a vancomycin-IRDye800CW conjugate (vanco-800CW) specific for Gram-positive bacteria.

**Results:**

38 synovial fluid samples were collected and analyzed. Significantly higher fluorescence levels were measured for *S. aureus*-positive samples compared to, respectively, other Gram-positive bacterial pathogens (*p* < 0.0001), Gram-negative bacterial pathogens (*p* = 0.0038) and non-infected samples (*p* = 0.0030), allowing a diagnosis of *S. aureus-*associated PJI within 2 h. Importantly, *S. aureus*-associated biofilms on extracted osteosynthesis materials from patients with FRI were accurately imaged with the AttoPolyT probe, allowing their correct distinction from biofilms formed by other Gram-positive bacteria detected with vanco-800CW within 15 min.

**Conclusion:**

The present study highlights the potential clinical value of the AttoPolyT probe for fast and accurate detection of *S. aureus* infection in synovial fluids and biofilms on extracted osteosynthesis materials.

**Supplementary Information:**

The online version contains supplementary material available at 10.1007/s00259-023-06499-4.

## Introduction

*Staphylococcus aureus* is an important and dreaded bacterial pathogen in orthopedic and trauma surgery. It is the causative agent in approximately 52% [1] and 27% [2] of septic arthritis and periprosthetic joint infections (PJIs), respectively, and in approximately 31% [3] of fracture-related infections (FRI) of osteosynthesis devices after surgical fracture treatment. Due to its high virulence and ability to evade the host-immune responses, the morbidity, mortality, and costs for healthcare systems due to *S. aureus* infections are very high compared to infections caused by other pathogens [4]. Moreover, effective treatment with antibiotics is hampered by the ability of *S. aureus* to form biofilms with restricted permeability and persistent bacteria on the implants [5]. As a consequence, infected implants need to be surgically debrided, exchanged, or permanently removed, and patients are subsequently treated with prolonged antibiotic therapy [6].

Most of the current culture-based and molecular methods for infection diagnosis and pathogen identification are time-consuming, whereas early diagnosis would contribute to better clinical results and faster adjustment of empirical antibiotic therapy. The latter is becoming increasingly relevant as, depending on demographics, resistance rates towards methicillin by *S. aureus* (MRSA) can be as high as 46% [7]. Culturing of synovial fluid and tissue surrounding the implants is nowadays considered the diagnostic gold standard, but time-to-positivity of *S. aureus* (joint) infections may take up to 2–5 days [8], during which uncertainty remains about the optimal treatment. Ideally, a definitive diagnosis of infection by the most common and impactful pathogen is obtained within hours after aspiration of synovial fluid in PJI, or alternatively, in real time while performing the infection revision surgery, as this facilitates an immediate decision on the most appropriate surgical and antimicrobial treatments.

**Fig. 1:**
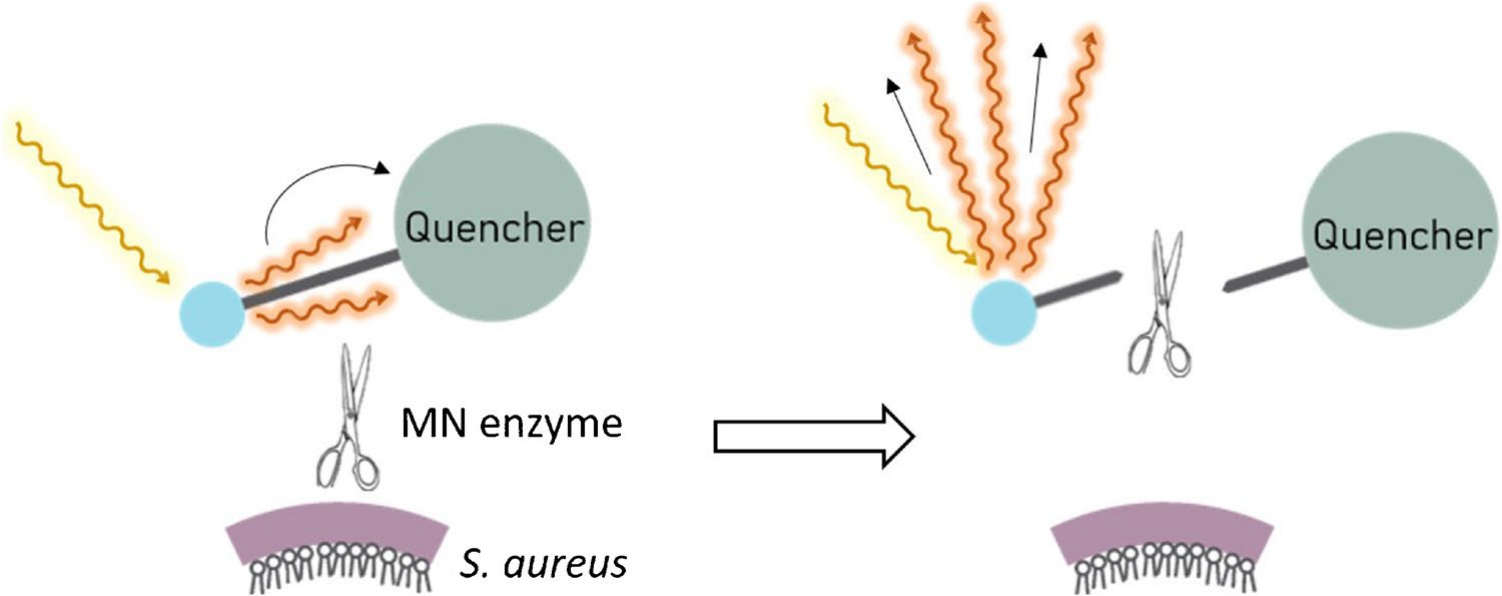
Nuclease-mediated activation of the AttoPolyT probe. The AttoPolyT probe is specifically cleaved by the secreted *S. aureus* micrococcal nuclease (MN) enzyme. This leads to a separation of the quencher from the fluorophore and, consequently, emission of fluorescence (515–535 nm, red arrows) upon excitation in the 490 nm Cy2 range (yellow arrow)

The present proof-of-principle study was aimed at investigating the diagnostic performance of the fluorogenic “AttoPolyT” probe, which is a “smart-activatable” optical probe that readily emits fluorescence after being cleaved by the micrococcal nuclease, a *S. aureus-*specific secreted enzyme (Fig. 1) [9–11]. Previous studies have already shown the potential of this and similar probes for accurate, fast (results within 2 h after obtaining the sample), and highly sensitive detection of *S. aureus* infection in mice [10], clinical blood [11], and blood culture samples [12]. The particularly high sensitivity of the AttoPolyT probe for micrococcal nuclease and its specificity for *S. aureus* versus relevant off-target bacterial species was previously demonstrated [11]. Moreover, due to their high signal-to-background ratio’s, specific imaging of *S. aureus* biofilms with probes like the AttoPolyT probe is theoretically feasible but had not been reported so far [10]. Here, we report the application potential of the AttoPolyT probe for the detection of *S. aureus* in clinical synovial fluid samples within 2 h and the first clinical application of *S. aureus*-specific biofilm imaging on extracted infected osteosynthesis materials (OSMs).

## Material and methods

### Nuclease-activated fluorescent AttoPolyT 4mer probe

The AttoPolyT oligonucleotide probe was synthesized, purified, and lyophilized by Integrated DNA Technologies Inc. (Coralville, IA USA). This probe is composed of ATTO488-TTTT-ZEN-IAbRQ (from 5′- to 3′-), where ATTO488 is the ATTO488 fluorophore, T is the deoxythymidine nucleotide, ZEN is IDT’s ZEN quencher, and IAbRQ is IDT’s Iowa Black RQ dark quencher. Upon receipt, the lyophilized probe was dissolved in TE-buffer (10 mM Tris; 0.1 mM EDTA; pH 8.0) to a final concentration of 391.6 µM, and the solution was then aliquoted into 1 µL stocks that were stored at − 80 °C until use. The AttoPolyT probe was received from Integrated DNA Technologies in July of 2019, and it was shown to remain stable when stored lyophilized at room temperature or frozen in aqueous solutions.

### Synovial fluid assay with the AttoPolyT probe

Synovial fluid samples from patients with a clinical suspicion of a (prosthetic) joint infection (i.e. a warm, painful, and swollen joint) were prospectively collected in the period from 01–03-2021 until 01–11-2022 at the University Medical Center Groningen (UMCG), and retrospectively from our existing synovial fluid biobank where the samples were stored at -80 °C [13]. The inclusion criteria for the present study were as follows: (1) patients had synovial fluid aspirated under aseptic conditions by arthrocentesis or intraoperatively, and (2) at least 1 mL of sample was available for the assay. There were no exclusion criteria. After the one-year study period, no more culture-negative samples were collected, because a sufficient number of samples had been obtained. All samples were cultured by standard microbiological work-up for pathogen identification (including Lancefield grouping for streptococci).

For the AttoPolyT assay, 1 mL of synovial fluid was spiked with 40 mM CaCl_2_ to a final concentration of 10 mM and heat-treated for 20 min at 90 °C to inactivate potential heat-sensitive micrococcal nuclease-inhibitory antibodies [11]. The solution was then centrifuged for 15 min at 16,000 RPM to obtain a clear supernatant fraction for the assay. 1 µL AttoPolyT probe stock solutions were diluted in 9 µL reaction buffer (50 mM Tris–HCl pH 9.0, 10 mM CaCl_2_) to a final concentration of 39.2 µM. 5 µL synovial supernatant fractions were then incubated in duplicate with 1 µL AttoPolyT probe in the presence of 4 µL reaction buffer and incubated for 1 h at 37 °C. To measure general background fluorescence and background fluorescence due to uncleaved AttoPolyT probe, 5 µL synovial supernatant fractions in 5µL reaction buffer without added probe, and 1 µL probe alone in 9 µL reaction buffer were incubated for 1 h at 37 °C. Then, 290 µL reaction stop buffer (50 mM EDTA in Dulbecco’s phosphate-buffered saline (DPBS) − / −) was added to all groups to block the activity of the micrococcal nuclease, samples were aliquoted in triplicate in a black flat-bottomed polystyrene 96-wells plate, and fluorescence was measured in a microplate reader (Biotek Synergy 2.0, BioTek Instruments, Inc., USA) using appropriate settings (excitation, 480 nm; emission, 520 nm).

**Fig. 2:**
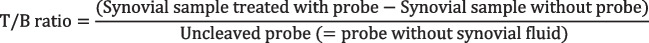
Formula applied to calculate the Target-to-Background (*T*/*B*) ratio for micrococcal nuclease activity in synovial fluid using the AttoPolyT probe assay

Average target-to-background (*T*/*B*) ratios were calculated by subtracting background fluorescence of the synovial fluid from the fluorescence of synovial fluid samples incubated with the AttoPolyT probe, and subsequent division by the fluorescence of the uncleaved probe (Fig. 2). Graphs were plotted in GraphPad Prism 8.1.1 (GraphPad Software, CA, USA). Statistical differences between (1) *S. aureus*-infected samples, (2) samples infected by other Gram-positive bacteria, (3) samples infected by Gram-negative bacteria, and (4) samples without detectable bacteria were calculated using the Kruskal–Wallis H test and the Dunn’s test in GraphPad Prism 8.1.1 and the Statistical Package for the Social Sciences (SPSS version 28, IBM Corp, USA).

### Detection and imaging of *S. aureus* biofilms using the AttoPolyT probe

#### In vitro fluorescence imaging of *S. aureus* biofilms on glass coverslips

To test whether the AttoPolyT probe can also be applied to detect and image *S. aureus* biofilms, we first performed in vitro experiments with lab-grown biofilms on 16 mm glass coverslips. A clinical isolate of *S. aureus* was grown overnight in Tryptic Soy Broth (TSB) and incubated in the presence of a sterile coverslip at an optical density (OD) of 0.1 in TSB enriched with 4% NaCl and 5% glucose. Biofilms were grown on the coverslips in 24 h at 37 °C. Coverslips for control groups were incubated in enriched TSB medium without bacteria. All coverslips were washed after 24 h with phosphate-buffered saline (PBS) to remove planktonic bacteria and incubated for 1 h with: (1) AttoPolyT probe in the presence of reaction buffer (yielding a final volume of 100 µL and concentration of 3.9 µM probe), (2) 100 µL reaction buffer alone, or (3) 100 µL PBS. Fluorescence images were recorded using an Amersham™ Typhoon 5 Biomolecular Imager (Cy2 filter 525BP20, wavelength 515–535 nm, photo-multiplier tube (PMT): 461). Images were analyzed using ImageJ software (National Institutes of Health, MD, USA); regions of interest (ROIs) were drawn around the coverslips for measurement of fluorescence intensity. Raw images were converted into color using the “Fire” Lookup Table setting in ImageJ. To verify the presence of biofilms on the coverslips, parallel to the experiments above, coverslips were incubated with 0.2 mg/mL Congo Red (which stains the Extracellular Polymeric Substance of biofilms [14]) and were also grown for 24 h at 37 °C.

#### Ex vivo multispectral fluorescence imaging of bacterial biofilms on extracted osteosynthesis materials using the AttoPolyT probe and the near-infrared vancomycin-IRDye800CW tracer

To extend our investigations with the AttoPolyT probe to clinical biofilms, we also imaged OSMs that had surgically been extracted from trauma patients at the UMCG for septic or a-septic reasons. For identification of pathogens in the biofilms, these OSMs were divided during surgery, using a nipper in case of osteosynthesis plates. Half of the material was used for standard culture-based microbiological work-up at the UMCG’s microbiological diagnostics laboratory, and the other half was used for research. For the present experiments, we also used extracted OSMs that were previously incubated with a conjugate of the antibiotic vancomycin and the near-infrared (NIR) dye IRDye800CW (in short vanco-800CW) as described by López‑Álvarez et al. [15]. This NIR fluorescence tracer binds specifically to the cell wall of Gram-positive bacteria. The chemical synthesis and application of vanco-800CW were previously described in close detail by van Oosten et al. [16]. Briefly, the implants were washed in PBS, incubated in 0.14 nmol/mL vanco-800CW for 15 min at 37 °C, and washed twice with PBS to remove the unbound tracer. The OSMs were stored at 4 °C in sterile bags until use for the present study. At the start of this study, all OSMs were washed with PBS and, subsequently, topically incubated with 3.9 µM AttoPolyT probe (final concentration) in reaction buffer for 15 min at 37 °C. This probe concentration was selected based on our observations in previous studies with the AttoPolyT and related probes, where it allowed strong probe performance in aqueous-phase reactions [11, 12]. The volume of the administered probe was adjusted to the size of the respective OSM. Prior and after incubation with the AttoPolyT probe, the OSMs were imaged with an Amersham™ Typhoon 5 Biomolecular Imager using appropriate wavelength filters for the AttoPolyT probe (Cy2: 515–535 nm, PMT 400). In addition, the OSMs that had also been incubated with vanco-800CW were imaged with appropriate NIR wavelength filters (IRLong: 810–840 nm, PMT 728). Fluorescence images were analyzed using ImageJ software. Raw images were converted into color using the “Fire” Lookup Table setting in ImageJ.

## Results

### Detection of *S. aureus* in synovial fluid with the AttoPolyT probe

A total of 38 synovial fluid samples were collected and analyzed. These samples could be divided into 4 groups: 13 samples were tested culture-positive with *S. aureus*, 13 samples were tested culture-positive with other Gram-positive bacteria (including *Staphylococcus epidermidis* (*n* = 3), *Streptococcus dysgalactiae* (*n* = 3), *Cutibacterium acnes* (*n* = 2), *Staphylococcus capitis*, *Streptococcus agalactiae (group B)*, *Streptococcus sanguis*, *Enterococcus faecalis*, and *Parvimonas micra*), and 4 samples were tested culture-positive with Gram-negative bacteria (including *Escherichia coli*, *Campylobacter jejuni*, *Enterobacter cloacae complex*, and *Stenotrophomonas maltophilia*). From 8 samples, no microorganisms could be isolated.

**Fig. 3:**
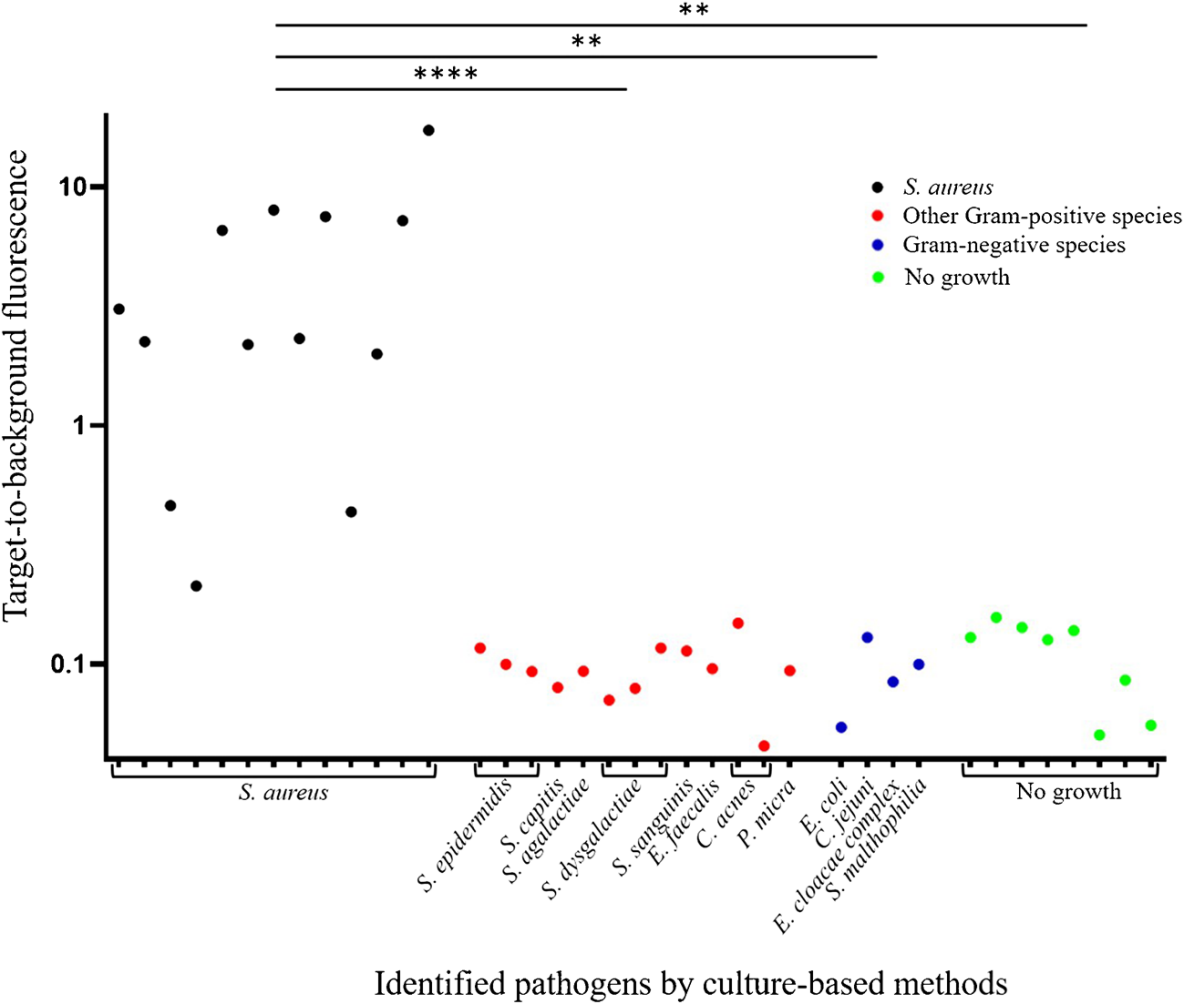
Fluorescence target-to-background ratios of nuclease assays with the AttoPolyT probe on synovial fluids from patients with suspected (prosthetic) joint infections. A significant difference was observed in the optical intensities measured in *T*/*B* ratios for synovial fluid samples containing *S. aureus* (*n* = 13) or other Gram-positive bacterial pathogens (*n* = 13; ****, *p* < 0.0001), as well as samples containing Gram-negative bacterial pathogens (*n* = 4; **, *p* < 0.01), or non-infected samples (“no growth”; *n* = 8, *p* < 0.01)

The results of the AttoPolyT probe assays with synovial fluids are shown in Fig. 3, and the respective numbers are presented in Supplementary Table S1. A Kruskal–Wallis *H* analysis showed that there was a statistically significant difference between the optical intensities measured in *T*/*B* ratio’s for the four sample groups (*χ*^2^ = 25.64, *p* < 0.001), whereas a Dunn’s post hoc analysis revealed that there was also a significant difference between the optical intensities measured in *T*/*B* ratio’s for culture-positive samples with *S. aureus* and, respectively, other Gram-positive bacterial pathogens (mean rank difference 20.12, *p* < 0.0001), Gram-negative pathogens (mean rank difference 20.50, *p* = 0.0038), and non-infected samples (mean rank difference 16.44, *p* = 0.0030). Moreover, the mean optical intensity of the *S. aureus*-infected group (mean *T*/*B* ratio, 4.6 ± 4.8; range, 0.2–17.4) was higher in comparison to samples containing other Gram-positive bacteria (mean *T*/*B* ratio, 0.1 ± 0.03; range, 0.05–0.15), Gram-negative bacteria (mean *T*/*B* ratio, 0.1 ± 0.03; range, 0.05–0.13), and sterile samples (mean *T*/*B* ratio, 0.1 ± 0.04; range, 0.05–0.16).

### Detection and imaging of *S. aureus* biofilms using the AttoPolyT probe

#### In vitro fluorescence imaging of *S. aureus* biofilms on glass coverslips

**Fig. 4:**
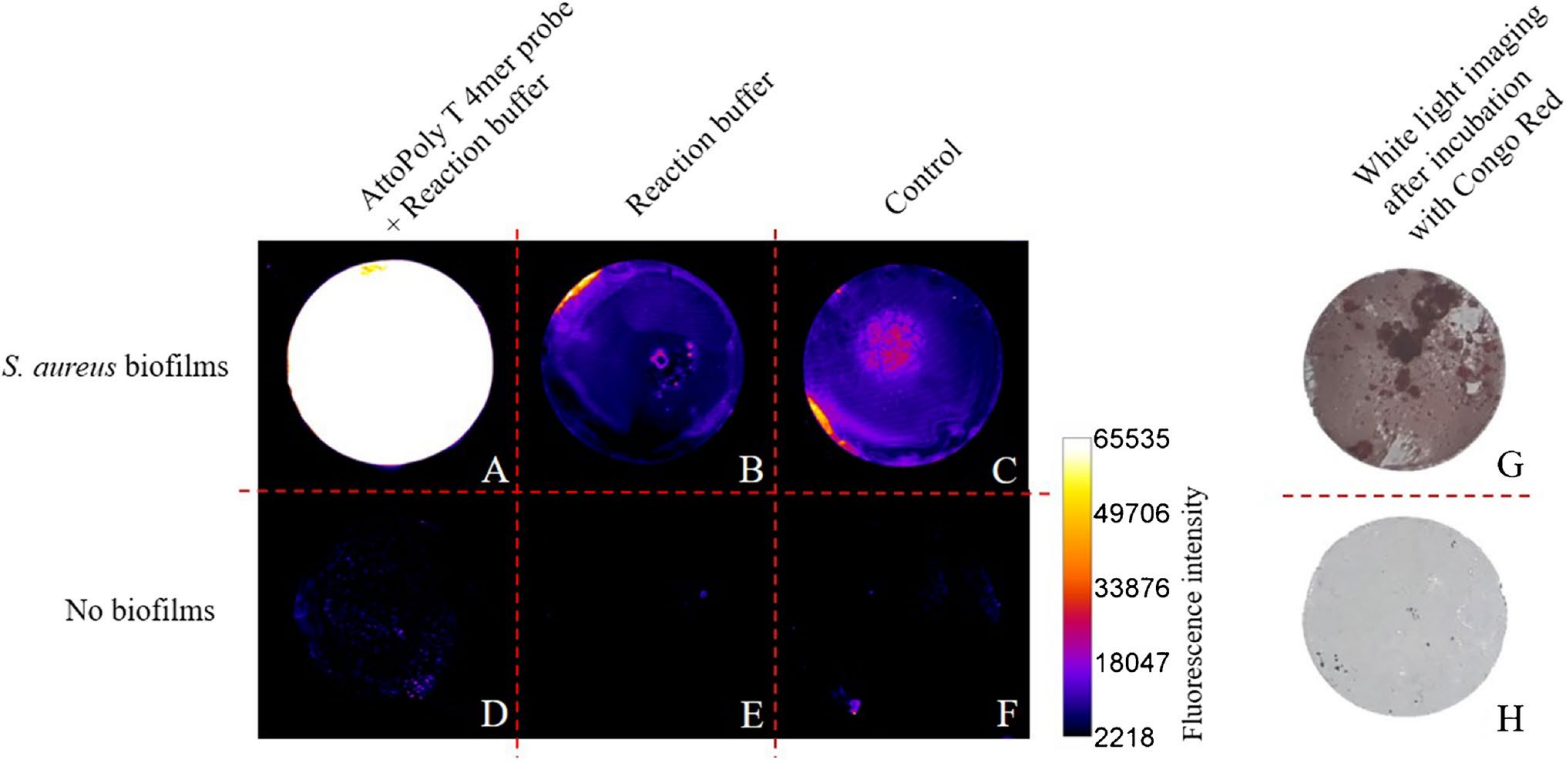
Imaging of in vitro-grown *S. aureus* biofilms on glass coverslips using the AttoPolyT probe. After incubation with or without the probe for 1 h, fluorescence was recorded using an Amersham™ Typhoon 5 Biomolecular Imager using the Cy2 filter. **A**–**C** Glass slides with *S. aureus* biofilm. **D**–**F** Glass slides without adherent bacteria. **A, D** Incubation with reaction buffer containing 3.9 µM AttoPolyT probe. **B, E** Incubation with reaction buffer alone. **C, F** Incubation with PBS. A clear difference is observed in the intensity of the emitted fluorescence between the *S. aureus* biofilms incubated with the **A** AttoPolyT probe and **B–F** the control groups. White-light imaging after parallel incubation of the coverslips with Congo Red confirms the **G** presence or **H** absence of *S. aureus* biofilms on the coverslips

To test whether the AttoPolyT probe can also be applied to detect *S. aureus* biofilms, we first performed experiments with in vitro-grown biofilms on glass coverslips. The imaging results of the coverslips are shown in Fig. 4. Notably, a clear difference was observed in the intensity of the emitted fluorescence between the *S. aureus* biofilms incubated with the AttoPolyT probe (Fig. 4A) and the control groups (Fig. 4B–F) that emitted substantially lower fluorescence signals. Comparison of the different ROIs revealed a 35-fold higher optical intensity for *S. aureus* biofilms treated with the AttoPolyT probe (mean, 65,129 counts) in comparison to coverslips incubated with probe but without biofilms (mean, 1855 counts), and a ninefold higher optical intensity in comparison to *S. aureus* biofilms incubated with reaction buffer alone (mean, 7205 counts). White-light imaging of the coverslips that had been incubated with Congo Red confirmed the presence or absence of *S. aureus* biofilms on the coverslips (Fig. 4G, H).

#### Ex vivo multispectral fluorescence imaging of bacterial biofilms on extracted osteosynthesis materials using the AttoPolyT probe and the near-infrared vancomycin-IRDye800CW tracer

OSMs from eight patients were collected during revision surgery at the University Medical Center Groningen in the period between January 2018 and August 2023. OSMs of six patients were incubated with vanco-800CW [15] prior to testing with the AttoPolyT probe. The OSMs of two other patients were exclusively used for testing with the AttoPolyT probe to exclude possible artefacts due to the prior incubation with vanco-800CW. Five of the respective patients had a clinical suspicion for a FRI, whereas the three other patients underwent revision surgery for non-septic reasons (negative controls) (Table 1). Routine diagnostic sonication and culturing of the extracted OSMs confirmed the presence of bacteria on the implants of patients 1, 2, 3, 4, and 7, whereas the cultures of patients 5, 6, and 8 remained culture-negative.

**Table 1:** Characteristics of included patients whose osteosynthesis materials (OSMs) had to be surgically extracted for suspected infections or a-septic causes

Patient	OSM in situ	Suspicion of infection?	Clinical signs of infection	OSM culture results
1	Fibula plate with screws after complicated ankle fracture	Yes	Warm ankle, progressive pain, elevated inflammation markers	***Staphylococcus aureus***
2	Tibia plate with screws after distal tibial fracture	Yes	Wound dehiscence, partially exposed implant	***Staphylococcus aureus*** *Enterococcus faecalis*
3	Tibia plate with screws after crural fracture	Yes	Wound dehiscence, partially exposed implant	*Acinetobacter radioresistens* *Citrobacter koseri* *Corynebacterium* spp. *Dermabacter hominis* *Staphylococcus haemolyticus*
4	Femur plate with screws after multiple non-union surgeries	Yes	No clinical signs, however on X-ray non-union at fracture site with implant failure femur plate	*Cutibacterium acnes*
5	Fibula plate with screws after Weber-C fracture	No	None, routine removal of lag screw	Negative
6	K-wires in carpalia after carpal fractures	No	None, routine removal of K-wires	Negative
7	Femur cerclages after femur gunshot wound and femur fracture	Yes	Fistula	***Staphylococcus aureus***
8	Tibia and fibula plate with screws after crural fracture	No	None, routine removal of tibial plate and screws	Negative

**Fig. 5:**
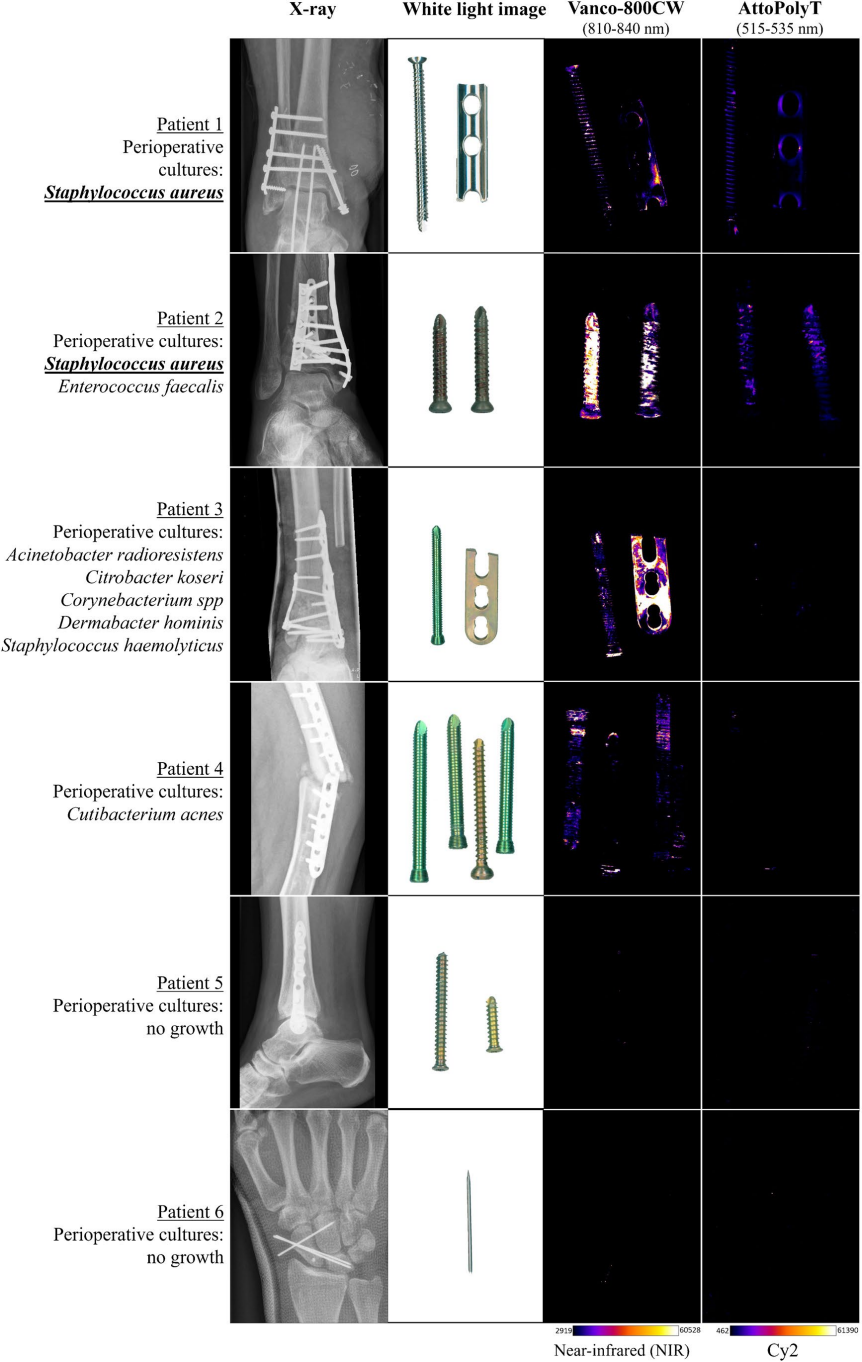
Ex vivo multispectral imaging of biofilms on patient-derived osteosynthesis materials (OSMs) with vanco-800CW and the AttoPolyT probe. OSMs that had tested either culture-positive or culture-negative were incubated with the AttoPolyT and vanco-800CW probes. Fluorescence images were recorded with an Amersham™ Typhoon 5 Biomolecular Imager using the Cy2 and IRLong filters to detect fluorescence of the AttoPolyT probe and vanco-800CW, respectively. Fluorescence signal was observed in both the Cy2 and IRLong range for OSMs from patients 1 and 2, who presented *S. aureus*-positive OSM cultures. In contrast, only fluorescence signal in the IRLong range was observed for OSMs from patients 3 and 4, who presented Gram-positive bacterial OSM cultures that did not include *S. aureus*. There were no fluorescence signals detectable for OSMs from patients 5 and 6, which had tested culture-negative

**Fig. 6:**
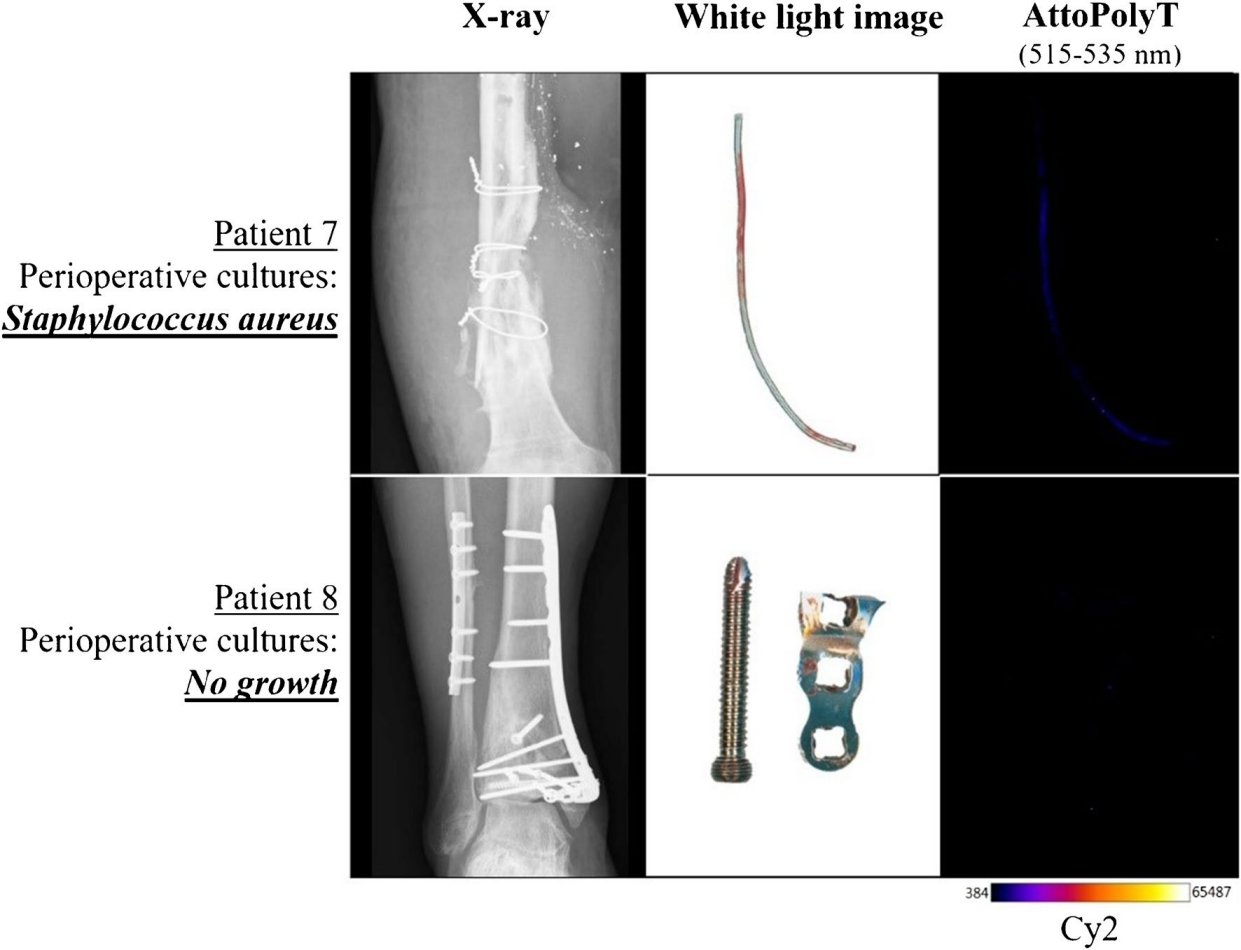
Ex vivo imaging of *S. aureus* biofilms on patient-derived osteosynthesis materials (OSMs) with the AttoPolyT probe. OSMs that had tested either culture-positive for *S. aureus* or culture-negative were incubated with the AttoPolyT probe. Fluorescence images were recorded with an Amersham™ Typhoon 5 Biomolecular Imager using the Cy2 filters. Fluorescence signal was observed in the Cy2 range for OSM from patient 7, who presented *S. aureus*-positive OSM cultures. There were no fluorescence signals detectable for OSMs from patient 8, who had tested culture-negative

The results of the imaging procedures with the extracted OSMs that were first incubated with vanco-800CW and subsequently with the AttoPolyT probe are shown in Fig. 5. The results of the extracted OSMs that were incubated only with the AttoPolyT probe are shown in Fig. 6. Irrespective of the prior incubation with vanco-800CW, there was no AttoPolyT-specific fluorescence signal detectable compared to the background (Supplemental Figure S1). After 15-min incubation with both compounds, clear fluorescence signals were detected in both the Cy2 and NIR ranges on the extracted OSMs of patients 1 and 2, meaning that vanco-800CW detected the presence of Gram-positive bacteria on these OSMs and that the AttoPolyT probe correctly identified *S. aureus* on these materials (Fig. 5). In addition*, S. aureus* was also correctly identified on the OSMs of patient 7, which was incubated only with the AttoPolyT probe (Fig. 6). On the OSMs of patients 3 and 4, only vanco-800CW fluorescence signals in the NIR range were detected. This was consistent with the presence of Gram-positive bacteria, but absence of *S. aureus*, as evidenced by diagnostic culturing. On the OSMs of patients 5, 6, and 8 (Figs. 5 and 6), no fluorescence signals were detected compared to the background, which is consistent with the observation that these OSMs remained culture-negative.

## Discussion

The results of this study demonstrate the versatility of the fluorogenic AttoPolyT probe for rapid detection of *S. aureus* in synovial fluid samples and biofilms on extracted OSMs. Our observations thus encourage the future implementation of novel molecular diagnostic approaches in clinical practice. These may include the rapid detection of *S. aureus* in synovial fluid within 2 h after obtaining the sample, non-invasive early detection, and imaging through the human skin of *S. aureus-*infected implants that were pre-coated with the AttoPolyT probe and in vivo intra-operative biofilm imaging after topical (local) administration of the AttoPolyT probe in the surgical area to “enlighten” clinical decision-making. In future applications, real-time intra-operative visualization of infection may, for example, guide decisions on the extraction of implants, or it may be used to determine whether an *S. aureus*-containing biofilm has successfully been removed during a debridement and implant retention (DAIR) procedure. These innovative fluorescence-guided surgical applications would require an appropriate intra-operative fluorescence camera system. Several of such systems have been developed to measure NIR fluorescence, and they have been used for clinical studies. On the other hand, few intra-operative fluorescence camera systems are available to measure green (~ 500 nm) fluorescent light [17, 18]. Such novel approaches stand in stark contrast to the current culture-based methods for infection detection, which are usually time-consuming and based on the recovery of actively dividing bacterial cells, often delaying optimal treatment [19]. Current molecular methods for infection diagnosis, such as PCR, may compete with the rapidity of the AttoPolyT probe results [13], but they are expensive, usually require specific expertise and equipment, and cannot be used for intra-operative purposes. The multispectral imaging approach described here, where two fluorescent probes with different targets were simultaneously imaged has, to the best of our knowledge, not yet been performed with patient-derived materials. We believe that this is a meaningful innovation, since it allows both for the detection of Gram-positive bacterial biofilms and the distinction of *S. aureus*-positive and -negative biofilms, which is important diagnostic information, in particular in regions of the world where MRSA is prevalent.

Previous studies used DNA-based fluorogenic probes similar to the AttoPolyT probe for detection of *S. aureus* in patient plasma [11] and blood cultures [12]. The results were comparable to those obtained in our present synovial fluid assay, although the differences in *T*/*B* ratios were larger between *S. aureus*-infected samples and *S. aureus*-negative samples in blood cultures. A plausible explanation for this discrepancy could be that the bacterial load is generally lower in synovial fluid of infected joints than in blood cultures where the bacteria are able to grow after the collection of the blood from patients with a suspected bacteremia. Moreover, the *T*/*B* fluorescence levels of *S. aureus-*infected samples were more diverse in our present study. This can be explained by the fact that the bacterial load in septic arthritis of (prosthetic) joints may vary significantly between patients, depending on the severity of the infection and the duration of the infection before a sample is obtained. Nonetheless, clear differences between *S. aureus*-infected synovial samples and other synovial sample groups were also observed in our present study, and the lowest *T*/*B* ratio of a *S. aureus*-infected sample (0.21) was still higher than the highest *T*/*B* ratio measured for samples without *S. aureus* (0.16).

Both the in vitro (Fig. 4) and the extracted OSM (Figs. 5 and 6) imaging results demonstrated that the high sensitivity provided by the AttoPolyT probe enabled detection of *S. aureus* even in the context of *S. aureus* biofilms, which are known to produce lower levels of micrococcal nuclease [20]. Of note, in the in vitro grown control biofilms that were not incubated with the AttoPolyT probe, we also observed low fluorescence signals in the Cy2 range (Fig. 4B, C). Likewise, the coverslips that carried no biofilms presented some minor fluorescent spots (Fig. 4D–F). We attribute this background to the autofluorescence of unidentified components in the TSB medium [21]. Despite the applied washing steps, this residual fluorescence was retained (in the biofilms) on the coverslips. A noteworthy observation made during the multispectral imaging of OSMs derived from patient 1, which was diagnosed for *S. aureus* carriage only, was that the fluorescence signals from the AttoPolyT probe and vanco-800CW did not completely co-localize (Fig. 5). This implies that these two compounds were spatially bound (vanco-800CW) or activated (AttoPolyT) at different sites on the OSMs. The reason for this difference is presently uncertain, but it most likely relates to the additional OSM washing and incubation steps required for the imaging with the two probes. Vanco-800CW specifically binds to the cell wall of Gram-positive bacteria which are tightly retained in the biofilm [16]. In contrast, the localization of the micrococcal nuclease and the digested AttoPolyT probe most likely depends on weak interactions of the nuclease and the ATTO488 fluorophore with components of the biofilm, which may be distinct from the vanco-800CW binding sites. Here, it should also be noted that the autofluorescence maxima of human tissue and the AttoPolyT probe are in the same range, whereas there is much less overlap for probes that fluoresce in the NIR range, like vanco-800CW [17, 22]. On the OSMs of patient 2, there was also less co-localization of both signals, but this is probably explained by the polymicrobial biofilm on the OSMs, which also carried the Gram-positive bacterium *E. faecalis* that will only bind vanco-800CW. Importantly, irrespective of the differences in signal localization and strength, the imaging results show that the OSMs from patients 1 and 2 were infected with *S. aureus*, a Gram-positive bacterial species that was also identified by diagnostic culturing of OSMs from these two patients.

A possible limitation of the present study is that relatively few samples with Gram-negative bacteria were included (*n* = 4) in the synovial fluid assay. This is due to the relatively low incidence of Gram-negative bacterial pathogens in (periprosthetic) joint infections [2]. Nonetheless, we still found a statistically significant difference between the sample group infected with Gram-negative bacteria and the group containing *S. aureus*. Another potential limitation of our study is that the imaging of screws as presented in Fig. 5 could be prone to some sampling bias, because bacterial biofilms are not perfectly homogeneously distributed on extracted OSMs [15]. Consequently, bacterial species may be identified on the OSMs used for culturing that are not necessarily also present on the OSMs inspected with vanco-800CW and the AttoPolyT probe. Furthermore, an important consideration to bear in mind when using the AttoPolyT probe is that a negative test result does not rule out infection by non-*S. aureus* bacterial species. This possible limitation can be circumvented by the combined use of multiple tracers or probes with different targets simultaneously, as exemplified in the present study by the combined use of the AttoPolyT probe and vanco-800CW. Also, antimicrobial susceptibility testing will remain an important part of the microbiological diagnostic procedures for optimal antimicrobial therapy and stewardship, as neither the AttoPolyT probe nor vanco-800CW provide information on the antimicrobial resistance of the detected bacteria.

In conclusion, our present study demonstrates the potential of the AttoPolyT probe for fast and accurate detection of *S. aureus* in synovial fluid. In addition, this probe allows real-time (multispectral) imaging of this most common and impactful pathogen on (extracted) OSMs. Bacteria-targeted infection imaging is currently an area that is still in its infancy, but the promising results of this and other studies highlight the value of adding novel molecular detection and imaging techniques to the present diagnostic toolbox for rapid and reliable diagnosis of orthopedic and trauma infections.

### Supplementary Information

Below is the link to the electronic supplementary material.

Supplementary file1 (PDF 514 KB)

## Data Availability

All data are provided with the manuscript, except the data that identify the included patients. The AttoPolyT probe was provided by Nuclease Probe Technologies, Inc., Lowell, MA, USA.
