# Correction to: A fluorogenic micrococcal nuclease‑based probe for fast detection and optical imaging of *Staphylococcus aureus* in prosthetic joint and fracture‑related infections

**DOI:** 10.1007/s00259-023-06538-0

**Published:** 2023-11-23

**Authors:** Jorrit W. A. Schoenmakers, Marina López‑Álvarez, Frank F. A. IJpma, Marjan Wouthuyzen‑Bakker, James O. McNamara, Marleen van Oosten, Paul C. Jutte, Jan Maarten van Dijl

**Affiliations:** 1 Department of Medical Microbiology and Infection Prevention, University of Groningen, University Medical Center Groningen (UMCG), Hanzeplein 1, 9700RB Groningen, The Netherlands; 2 Department of Orthopaedics, University of Groningen, University Medical Center Groningen (UMCG), Groningen, The Netherlands; 3 Department of Surgery, Division of Trauma Surgery, University of Groningen (UMCG), Groningen, The Netherlands; 4 Nuclease Probe Technologies, Inc, Lowell, MA USA

**Correction to: European Journal of Nuclear Medicine and Molecular Imaging**

**https://doi.org/10.1007/s00259-023-06499-4**

The authors regret that the formula for Figure 2 that appears in the original article was duplicated.

The original article has been corrected.

