# Supplementary material for: A fluorogenic micrococcal nuclease-based probe for fast detection and optical imaging of Staphylococcus aureus in prosthetic joint and fracture-related infections
Source: Eur J Nucl Med Mol Imaging. 2023 Nov 14;51(10):2988–97. doi: 10.1007/s00259-023-06499-4 (PMC11300479; doi:10.1007/s00259-023-06499-4)
Supplement: Supplementary file 1 — Supplementary file1 (PDF 514 KB) [file 259_2023_6499_MOESM1_ESM.pdf]

Supplementary Materials

Identified pathogen	T/B ratio
Staphylococcus aureus	3,086957
Staphylococcus aureus	2,253968
Staphylococcus aureus	0,462963
Staphylococcus aureus	0,212871
Staphylococcus aureus	6,599010
Staphylococcus aureus	2,193121
Staphylococcus aureus	8,019802
Staphylococcus aureus	2,321267
Staphylococcus aureus	7,524752
Staphylococcus aureus	0,436170
Staphylococcus aureus	2,000507
Staphylococcus aureus	7,244681
Staphylococcus aureus	17,35106
Staphylococcus epidermidis	0,116883
Staphylococcus epidermidis	0,100000
Staphylococcus epidermidis	0,093333
Staphylococcus capitis	0,080000
Streptococcus agalactiae (group B)	0,093506
Streptococcus dysgalactiae	0,070707
Streptococcus dysgalactiae	0,079208
Streptococcus dysgalactiae	0,117021
Streptococcus sanguinis	0,113861
Enterococcus faecalis	0,095745
Cutibacterium acnes	0,148936
Cutibacterium acnes	0,045455
Parvimonas micra	0,094059
Escherichia coli	0,054455
Campylobacter jejuni	0,129630
Enterobacter cloacae complex	0,084416
Stenotrophomonas maltophilia	0,100000
No growth	0,129630
No growth	0,157407
No growth	0,142857
No growth	0,126667
No growth	0,138614
No growth	0,050505
No growth	0,085859
No growth	0,055556
No growth	0,160000

Supplementary Table S1. Measured average fluorescence target-to-background ratios of the nuclease assays with the AttoPolyT probe on synovial fluids from patients with suspected (prosthetic) joint infections as presented in Figure 3. The listing of identified bacterial species and samples testing negative for bacterial growth ('no growth') from top to bottom corresponds with the graphical representation from left to right in Figure 3.

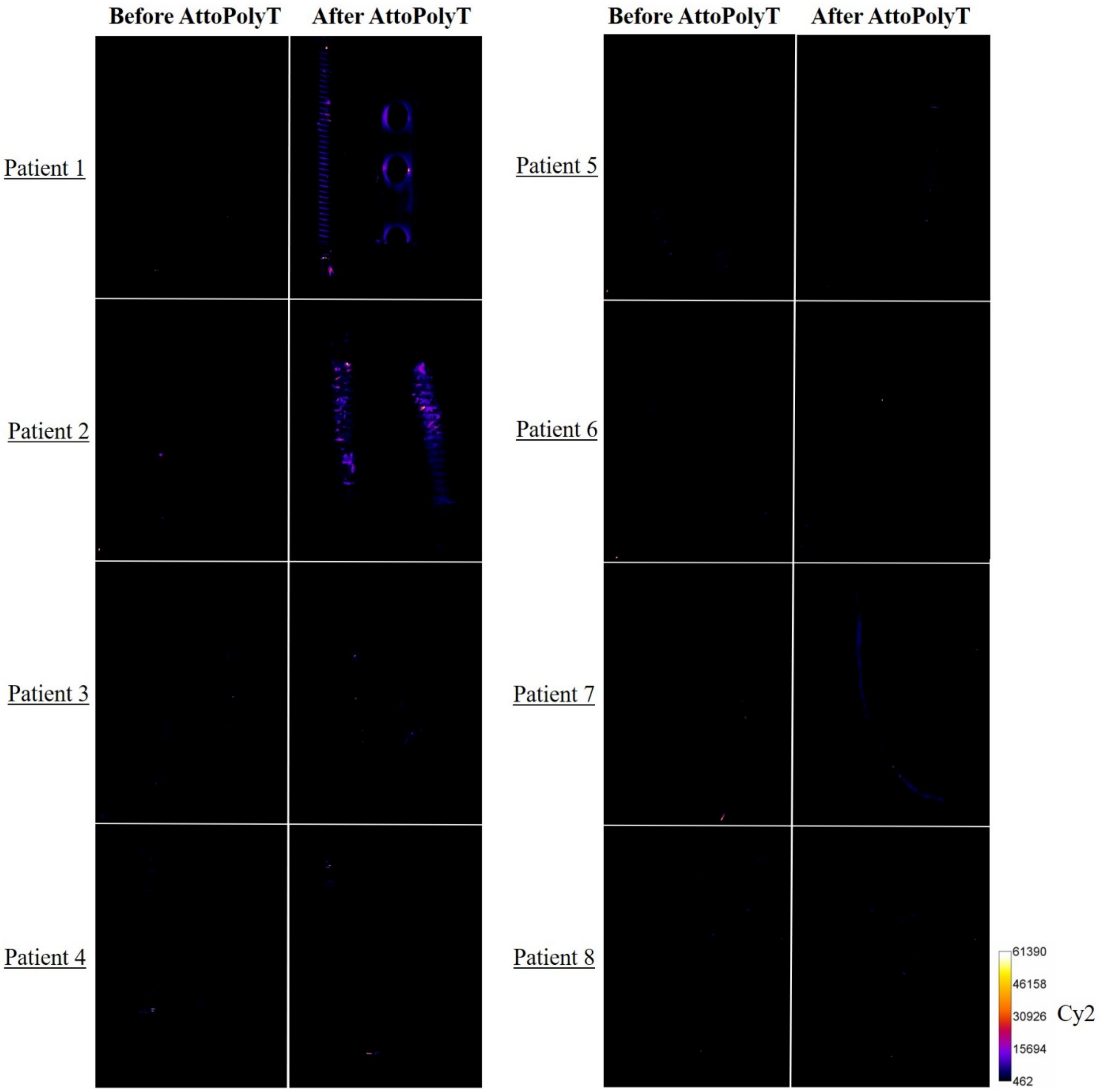

Supplemental Figure S1. Fluorescence images of extracted osteosynthesis materials (OSMs) before and after incubation with the AttoPolyT probe. Images captured with an Amersham™ Typhoon 5 Biomolecular Imager using the Cy2 filter, PMT 400. On the OSMs of patients 1, 2 and 7 *Staphylococcus aureus* was identified by culture, whereas on the OSMs of patients 3 and 4 other bacteria were identified. From the OSMs of patients 5, 6 and 8 no bacteria were cultured. Please note that the pre-incubation imaging of vancomycin-800CW was previously performed as described by López-Álvarez et al. (15)
